# Quantitative characterization of T-cell repertoire and biomarkers in kidney transplant rejection

**DOI:** 10.1186/s12882-016-0395-3

**Published:** 2016-11-21

**Authors:** Houda Alachkar, Martin Mutonga, Taigo Kato, Sowjanya Kalluri, Yoichi Kakuta, Motohide Uemura, Ryoichi Imamura, Norio Nonomura, Vikas Vujjini, Sami Alasfar, Hamid Rabb, Yusuke Nakamura, Nada Alachkar

**Affiliations:** 1 School of Pharmacy, University of Southern California, Los Angeles, CA 90089 USA; 2 Department of Medicine, University of Chicago, Chicago, IL 60637 USA; 3 Department of Medicine, Division of Nephrology, Johns Hopkins University School of Medicine, Baltimore, MD USA; 4 Department of Urology, Osaka University Graduate School of Medicine, Osaka, Japan; 5 Kendall regional medical center, Miami, FL 33175 USA; 6 Johns Hopkins Hospital, Baltimore, MD 21287 USA

**Keywords:** T-cell, Kidney transplant, T cell mediated rejection, T cell sequencing

## Abstract

**Background:**

T-cell-mediated rejection (TCMR) remains a major cause of kidney allograft failure. The characterization of T-cell repertoire in different immunological disorders has emerged recently as a novel tool with significant implications. We herein sought to characterize T-cell repertoire using next generation sequencing to diagnose TCMR.

**Methods:**

In this prospective study, we analyzed samples from 50 kidney transplant recipients. We collected blood and kidney transplant biopsy samples at sequential time points before and post transplant. We used next generation sequencing to characterize T-cell receptor (TCR) repertoire by using illumina miSeq on cDNA synthesized from RNA extracted from six patients’ samples. We also measured RNA expression levels of FOXP3, CD8, CD4, granzyme and perforin in blood samples from all 50 patients.

**Results:**

Seven patients developed TCMR during the first three months of the study. Out of six patients who had complete sets of blood and biopsy samples two had TCMR. We found an expansion of the TCR repertoire in blood at time of rejection when compared to that at pre-transplant or one-month post transplant. Patients with TCMR (*n =* 7) had significantly higher RNA expression levels of FOXP3, Perforin, Granzyme, CD4 and CD8 in blood samples than those with no TCMR (*n =* 43) (*P =* 0.02, *P =* 0.003, *P =* 0.002, *P =* 0.017, and *P =* 0.01, respectively).

**Conclusions:**

Our study provides a potential utilization of TCR clone kinetics analysis in the diagnosis of TCMR. This approach may allow for the identification of the expanded T-cell clones associated with the rejection and lead to potential noninvasive diagnosis and targeted therapies of TCMR.

## Background

Kidney transplantation remains the treatment of choice for patients with end stage renal disease (ESRD) and advanced chronic kidney disease (CKD). In spite of the improvement in short term allograft survival due to the newer potent immunosuppression [1] in the recent decades, long-term survival has not improved comparatively [2, 3]. Cumulative data have emphasized the importance of late clinical and sub-clinical rejections as major causes for shorter allograft survival and ultimately allograft loss. These types of rejections are, in most part, very difficult to treat and are often diagnosed very late after the onset of the rejection.

The diagnosis of acute kidney rejection still relies on obtaining kidney biopsy. In addition to the risks of this procedure, such as bleeding, obtaining kidney biopsy is costly and time consuming for the physicians, staff and patients. Therefore, research efforts have focused on identifying biomarkers that can be easily obtained from patient’s fluid (blood or urine), and can serve as markers for acute rejection [4–10]. However, the complexity of the immune system involved in rejection and the lack of specificity and sensitivity of these biomarkers limited their use in clinical settings [11].

T cell-mediated rejection (TCMR) is the most common cause of acute rejection [12], and is a major cause of allograft dysfunction and failure [13]. It is crucial to detect this type of rejection as early as possible, so it can be treated promptly before it leads to irreversible interstitial fibrosis and tubular atrophy (IFTA) [14]. In transplant patients, an allorecognition process occurs when T-cells, via their receptors (TCRs), recognize donor HLA molecules. This recognition is either direct when T-cells recognize intact donor HLA-peptide complex molecules presented by donor cells, or indirect when peptides derived from donor major histocompatibility complex (MHC) or minor Histocompatibility (miH) antigen are degraded and presented by recipient antigen presenting cells (APCs). Both direct and indirect allorecognition processes are thought to contribute to acute and chronic allograft injury [15]. Traditional methods for measurements of donor-reactive memory T-cells by flow cytometry and cytokine ELISPOT have shown to predict post transplant outcome [16–18]. While these seem to be promising biomarkers in transplantation, such measurement of T-cells requires an intensive labor and high cost, in addition to the complexity of the assays.

With the recent feasibility of high throughput sequencing, investigations have been directed toward a comprehensive analysis of TCR repertoire in the transplant setting. In this study, we utilized an mRNA-based, 5′RACE (rapid amplification of cDNA ends) PCR method followed by next generation sequencing (NGS) technology approach [19, 20] to identify combination of TCR beta (TCRB) in blood and graft samples obtained before and after kidney transplantation. We comprehensively characterized the detailed TCR repertoire changes in patients with kidney transplant in an attempt to correlate expansion of certain T-cell populations with graft rejection. We also measured the RNA expression levels of FOXP3, Perforin, Granzyme, CD4 and CD8 in blood samples to correlate these biomarkers with TCMR.

## Methods

### Study cohort

This is a prospective multi-center study approved by the Institutional Review Boards of Johns Hopkins Hospital, University of Chicago, and Osaka University. Patients were recruited after written consents at Johns Hopkins Hospital and Osaka University Hospital. We enrolled 50 patients who receive kidney transplantation between September 2013 and June 2014. Table 1 shows the clinical characteristics of our study cohort.

**Table 1:** Patients clinical characteristics

Patients parameters	*n =* 50	TCMR	No-TCMR	*P* value
Mean age (SD), years	51 (15)	45 (13)	52 (15)	0.25
Gender, male (%)	28 (56)	2 (28)	26 (60)	NS
Race (%)				
White	17 (34)	3 (43)	14 (33)	NS
Black	21 (42)	1 (14)	20 (46)	NS
Asian	7 (14)	3 (43)	4 (9)	NS
Others	5 (10)	0	5 (12)	NS
Preemptive (%)	8 (16)	1 (14)	7 (16)	NS
Mean CIT (SD) hours	19.7 (12.9)	12.5 (16.8)	20 (12.3)	0.22
Cause of kidney disease (%)				
HTN	8 (16)	1 (14)	7 (16)	NS
DM and HTN	16 (32)	2 (29)	14 (32)	NS
Glomerular diseases	17 (34)	3 (43)	14 (32)	NS
Others	10 (20)	1 (14)	9 (20)	NS
DGF	18 (36)	1 (14)	17 (39)	NS
Induction therapy (%)				
Thymoglobulin	36 (72)	4 (57)	32 (74.4)	NS
Alemtuzumab	7(14)	0	7 (16.2)	NS
Basiliximab	7 (14)	3 (43)	4 (9.3)	NS
Type of Donor (%)				
Living	16 (32)	4 (57)	12 (28)	NS
Deceased	34 (68)	3 (43)	31 (72)	NS

*TCMR* T cell mediated rejection, *SD* standard deviation, *NS* not significant, *CIT* cold ischemic time, *DM* diabetes mellitus, *HTN* hypertension, *DGF* delayed graft function

### Samples’ collection

We collected blood samples before kidney transplantation, at 24–48 h, one week, one month and three months after transplantation, and at time of confirmed TCMR. We also obtained kidney graft biopsy samples from the patients who had TCMR.

We collected 15 mL of blood sample from each patient at each time point in two Cell Preparation Tubes (CPT). These samples were processed in our laboratory within one hour of collection. After collecting the blood samples, we processed the samples using this centrifuging setting: temperature of 21 °C; speed of 3000 revolutions per minute (RPM), for 20 min with breaks on. After the first round of centrifugation we collected the cells carefully and wash them twice with PBS. We added PBS to fill the tube up to 15 mL and centrifuged it (temperature: 21 °C; speed: 1500 RPM; time: 10 min; Breaks on). We did a second wash by discarded the supernatant PBS and disturbed the cell pellet at the bottom by gentle tapping; we added 10 mL of PBS and centrifuged at the same setting. Finally, we collected the cell pellet in the small tube and centrifuged it for 2 min in the cold room to remove supernatant fluid, and preserved it at −80 °C until used for sequencing.

We used frozen section of the kidney biopsies and saved them in −80 °C. We extracted the RNA from these tissue samples.

### RNA isolation and PCR amplification

Total RNAs were isolated from peripheral blood mononuclear cells (PBMCs) and from graft biopsies using RNeasy mini kit (Qiagen, Valencia, CA) and treated with DNase to remove genomic DNA contamination. cDNA was then synthesized using the SMART cDNA library construction kit (Clontech Laboratories, Mountain View, CA), according to the manufacturer’s instructions. A common adaptor (SMART IV oligonucleotide) was ligated to the 5′ end of cDNA. PCR was then performed to amplify all the possible combination of TCR beta from cDNA, using one common forward primer, which is designed based on the sequence of SMART IV adaptor and a reverse primer specific to the constant region of TCR beta [21, 22].

### Template preparation and sequencing

Six patients had complete sets of blood and tissue samples at the intended time points; these samples were sequenced. Out of these six patients, two had TCMR. Each sample was barcoded at both ends of the library with different combination of index 1 and index 2 using Nextera XT kit (Illumina, San Diego, CA, US). Several samples were pooled together into single sequencing run on the Illumina MiSeq using Miseq Reagents Kits v3 600 cycles (Illumina).

Sequences analysis were performed using Tcrip algorithm [21, 22]. Sequencing reads in FASTQ files were mapped to the reference sequences derived from IMGT/GENE-DB (http://www.imgt.org), using Bowtie2 aligner (Version 2.1.0) [21, 22]. The V, D, J genes were designated according to the nomenclature provided by the international ImMunoGeneTics information system (IMGT). A CDR3 region was defined by identifying the second conserved cysteine encoded in the 3′ portion of the V segment and the conserved phenylalanine encoded in the 5′ portion of the J segment that form the boundaries of the CDR3. The nucleotide sequences between both conserved TCR V cysteine and TCR J phenylalanine were extracted to determine the amino acid sequence of CDR3 region.

### Analysis of TCMR biomarkers

To analyze markers of activated T-cells in kidney transplant patients and correlate these markers with graft rejection, we measured mRNA levels of the following genes: FOXP3, Perforin, Granzyme, CD4 and CD8 by RT-PCR in all blood samples of the whole cohort.

### Statistical analysis

The inverse Simpson’s index was calculated based on the following equation:

$$ {D}_S={\left[\frac{{\displaystyle {\sum}_{i=1}^K}{n}_i\left({n}_i-1\right)}{N\left(N-1\right)}\right]}^{-1} $$

Where *K* is the total number of clonotypes, *n* is the number of sequences belonging to the *i*-th type, and *N* is the total number of sequences for which clonotypes are determined [21, 22]. Paired Student’s *t*-test (two-tailed) was performed for comparison of total proportion of the most abundant ten CDR3 sequences or the diversity index between groups, using GraphPad Prism version 6.0. A *p* value of less than 0.05 was considered statistically significant.

## Results

### Variable, joining, and diversity gene segments (V(D)J) combination and CDR3 sequence analysis

Table 2 shows the clinical characteristics of all seven patients. Blood and tissue samples of the six patients, who had complete sets, were analyzed using a 5′ RACE PCR approach. We identified an average of 546,798 TCRB sequence reads in blood samples and 452,195 in graft samples. The observed sequence reads allowed us to identify the majority of the functional V exons in TCRB, indicating a good coverage of TCR gene by our cDNA sequencing approach. After defining V(D)J combinations for TCRB, we further defined individual CDR3 sequences using our newly developed algorithm. On average, we were able to identify 171,112 observed clones of 8,198 unique CDR3 sequences for TCRB in blood samples and 149,446 observed clones of 4510 unique CDR3 sequences for TCRB in graft samples.

**Table 2:** Clinical characteristics of all seven patient with TCMR

ID	Age, Y	Gender	Race	Type of transplant	Cause of ESRD	cPRA	DSA	Induction	Time to rejection (months)	Type of TCMR	Reason for rejection
1	60–65	Male	African American	DDRT	HTN	0	DR53 Flowcytometric cross match level	Thymoglobulin	19	2A	BK viremia/lower immunosuppression
2	30–35	Male	White	DDRT	Diabetic nephropathy	0	De novo DQA5 Below flowcytometric crossmatch	Thymoglobulin	11	1A	Lower dose of MMF
3	20–25	Female	White	LRT	IgA nephropathy	0	De novo DSA: DR7, DR53, DQA3, DQB2, and DQB7 positive cytotoxic crossmatch	Thymoglobulin	21	1B	
4	40–45	Female	White	LRT	Lupus nephritis	9%	Negative	Thymoglobulin	1	1A	Lower dose of MMF
5	45–50	Female	Asian	LRT	Unclear Etiology	10%	Negative	IL2 antagonist	3	1A	
6	55–60	Female	Asian	DDRT	Chronic glomerular nephropathy	0	Negative	IL2 antagonist	3	1A	
7	40–45	Female	Asian	LRT	Diabetic nephropathy	70%	Negative	IL2 antagonist	35	1A	

*cPRA* calculated Panel Reactive Antibodies, *DSA* Donor Specific Antibodies, TCMD T cell mediated rejection, *DDRT* deceased donor renal transplant, *HTN* hypertension, *MMF* mycophenolate mofetil, *LRT* living donor renal transplant

### Frequency of expanded CDR3 TCR unique clones

**Fig. 1:**
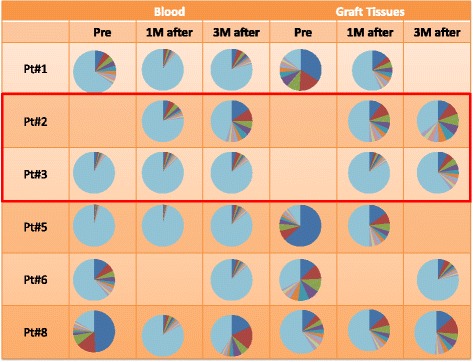
The clonality of T lymphocytes in blood and grafts of six kidney transplant patients. The distribution of the unique CDR3 sequences detected in TCRB. Each pie graph represents one sample, showing the frequencies of the ranked top ten clones and the light blue color represent sum of the frequencies of the remaining clones

Figure 1 shows the frequency of each of the top ten most abundant CDR3 unique clonotypes observed in blood and graft tissues of the six patients, indicating very strong enrichment of certain T-cell clones in some patients. The sum of the frequencies of the ten most abundant CDR3s in the six patients ranged from 4.6% to 83.5% (mean ± standard error (SE) = 26.1 ± 5.6%) and from 16.4% to 90.4% (mean ± SE = 52.7 ± 6.1%) for blood and graft, respectively.

### Expansion of TCR clones in repertoire of patients with graft rejection

**Fig. 2:**
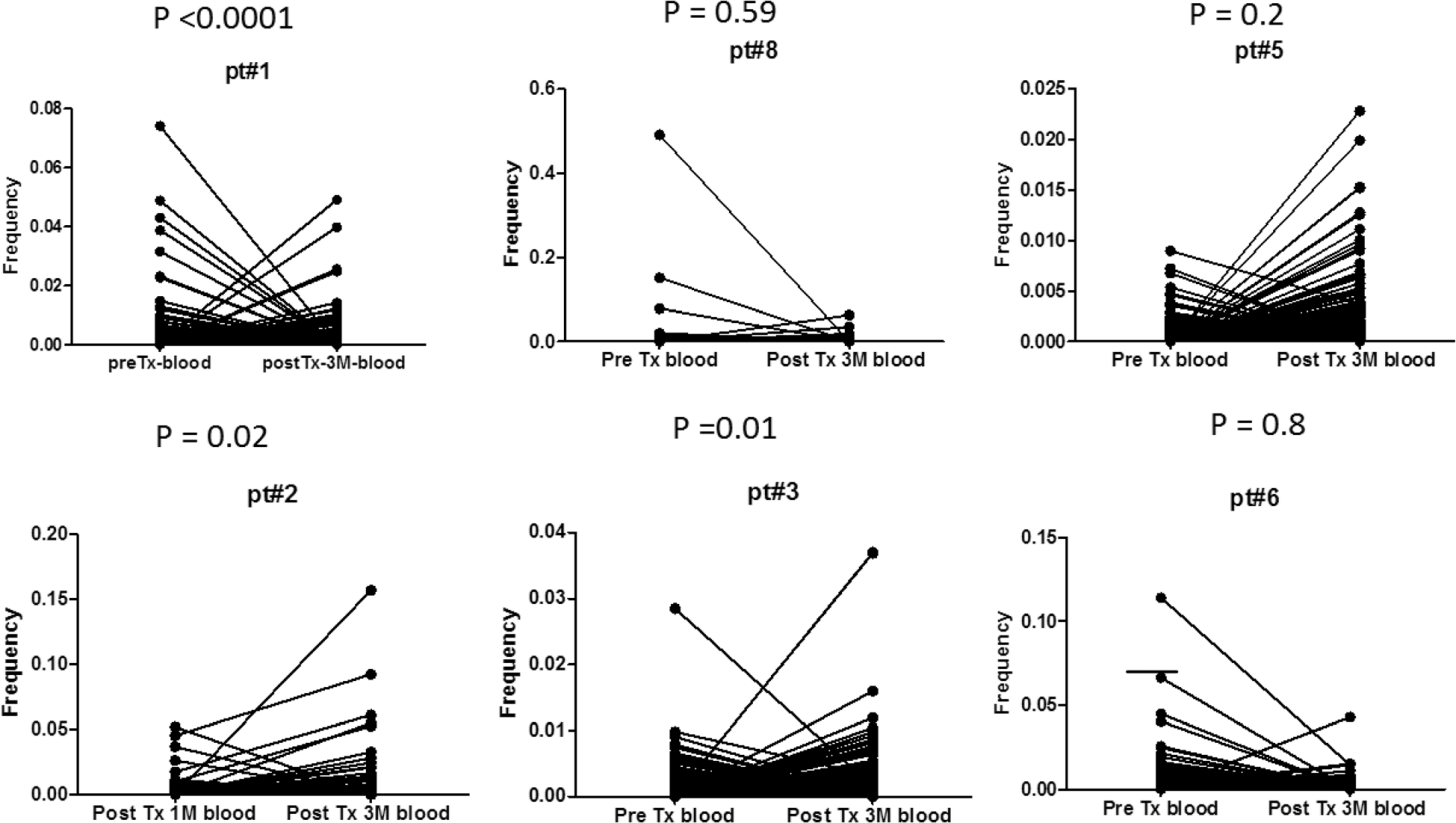
Expansion of TCR repertoire at the time of graft rejection. We generated the recurrent TCR repertoire by combining all TCR clones appeared at any time point (before and after transplant) in samples obtained from blood or graft. And then compared the frequency of each clone in the recurrent repertoire at the time of rejection (or the same time in case of patients with no rejection) with that at the earliest time point available for analysis (before transplant or 1 month post transplant)

To examine whether enrichment of certain T-cells may be involved in the development of graft rejection, we analyzed changes over time from baseline at the time of transplant and sequentially at one month and three months after transplant. On the basis of their V(D)J combination and defined CDR3 sequences, we sorted independent cDNA sequences according to their number of appearance in the sequence reads from the most to least abundant. We noted the ten most abundant CDR3 sequences at each time point. We combined the top ten clones that appeared at each time point and generated a recurrent CDR3 profile for each patient of all combined time points. We compared the recurrent CDR3 profile from blood samples obtained at the time of transplant with that from samples obtained at time of TCMR. We found that only the two patients with TCMR showed significant expansion of their recurrent CDR3 profiles (Fig. 2).

### Tracking TCRB clones in rejected graft back to their first appearance in blood or graft

**Fig. 3:**
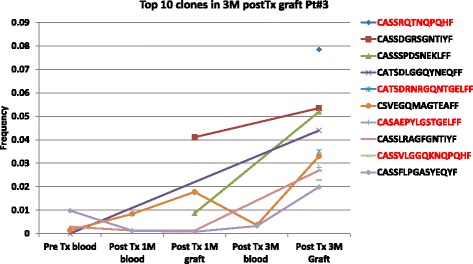
Tracking the top ten clones presented in the graft at the time of rejection to their earliest presence in blood or graft. We obtained the top ten most frequent clones observed in graft of patient #3 (TCMR), and showed the frequencies at which these clones were present in the blood and graft samples obtained at earlier time point

We tracked the top ten clones of the TCRB repertoire obtained from the graft tissues at time of TCMR, and we examined their presence in the repertoire obtained from blood and graft tissues at earlier time points (i.e.; 2 months and three months earlier). We found that among the top 10 clones, at least six clones were observed at earlier time points either in the blood or in the graft samples (Fig. 3).

### Diversity of TCR repertoire in blood and grafts

**Fig. 4:**
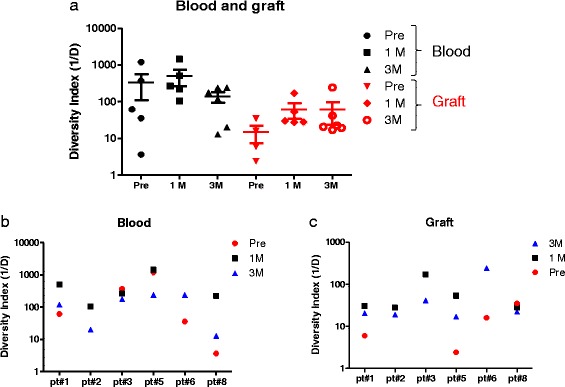
TCR repertoire diversity in blood kidney transplant patients. The diversity of the TCR repertoire was calculated for each sample using Simpson index. (**a**) Diversity indexes were compared between blood and graft samples. Diversity of TCR repertoire in blood samples (**b**) and graft (**c**) were compared between different patients

**Fig. 5:**
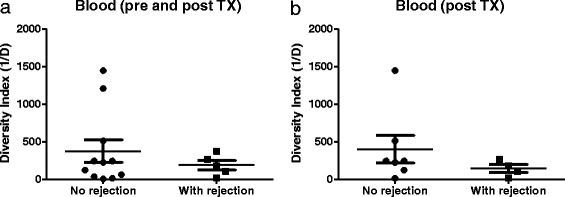
TCR repertoire diversity according to graft rejection status. The diversity of TCR repertoire of samples obtained from blood pre and post transplant (**a**) or post-transplant (**b**) was compared between patients with graft rejection and those without

To compare the TCR diversity among the six patients, we calculated the inverse Simpson’s diversity index (1/Ds) where higher values suggest a diversity increase of the TCR repertoire. TCR repertoire diversity was lower in graft samples compared to that in blood samples (Fig. 4a ). Three patients had a higher diversity of TCR repertoire in the blood at three months after transplants compared to that of samples obtained before transplant. On the other hand, in five patients, the TCR diversity was significantly lower at three months after transplant in comparison with that at one month after transplant (Fig. 4b). In patients of which we had graft samples pre and post-transplant (*n =* 4), we found that three patients showed an increase in the diversity of TCR repertoire in graft samples obtained three months after transplant in comparison with that obtained just before transplant. Interestingly, in five patients who had graft biopsy samples at 1 and 3 months after transplant, we found that the TCR repertoire was less diverse at three months post transplant compared to that at 1 month post-transplant (*P =* 0.003; Fig. 4c). No significant difference was found in the diversity of the TCRB repertoire between blood samples obtained from patients with TCMR and patients without TCMR, even when similar time points were compared (Fig. 5).

### Blood biomarkers of cell mediated rejection

**Fig. 6:**
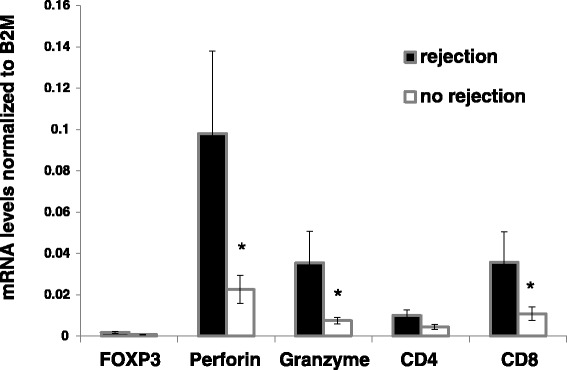
T-cells markers expression according to graft rejection status. The mRNA expression of T-cell markers (Perforin, Granzyme, CD4 and CD8) were measured in blood samples obtained from patients with graft rejection and those without rejection

We found that at any time point, blood samples of patients with TCMR (*n =* 7) had significantly higher levels of FOXP3, Perforin, Granzyme, CD4 and CD8, comparing to the 43 patients without rejection (*P =* 0.02, *P =* 0.003, *P =* 0.002, *P =* 0.02, and *P =* 0.01, respectively); Fig. 6.

## Discussion

T-cell immune response is a major cause of post kidney transplant complications. Although the introduction of newer potent immunosuppressive drugs in the last two decades resulted in significant decrease in early acute TCMR, late TCMR and chronic rejections remain common causes of allograft failure [23]. To prevent TCMR, patients require the right amount of immunosuppressions that keep the immune response adequately suppressed; yet it has the capacity to respond to pathological insults such as infections and malignant turnovers. Finding such balance has been a major goal and working progress by the transplant healthcare providers and investigators.

Our data herein, constitutes a very promising step toward identifying T-cell markers that can be utilized in the diagnosis of TCMR to enable clinicians to treat it promptly; and also to potentially provide the tools to individualize immunosuppressive therapy. We have previously applied NGS techniques to comprehensively analyze T-cell repertoire in blood samples from patients who underwent hematopoietic stem cell transplant [21]. We found that patients with graft versus host disease (GVHD) had a significant expansion of certain TCR clones and decrease in TCRB diversity in comparison with that in non-GVHD patients [21].

Our study demonstrated a large turnover of the T-cell populations over time consistent with conditioning immunosuppressive treatments that the patients received. Importantly, high correlation of the TCR repertoire was observed between blood and graft at the time of rejection. Although they cannot precisely point out clones responsible for the graft rejection, our data suggest the feasibility of tracking such clones once identified in the blood. We identified top ten clones profile or recurrent CDR3 profile, which included all the top clones that appeared at each time point in blood and graft samples. We found an expansion of the recurrent CDR3 profile from blood samples obtained at the time of rejection compared to earlier time points in patients with TCMR. Since this phenomenon was not observed in non-TCMR patients, it suggests that TCR clones that cause TCMR could be tracked to earlier time points; therefore identification of such clones can possibly provide a diagnostic value.

The utility of T-cell sequencing in the transplant field has been increasingly investigated. A recent study assessed kidney allograft dysfunction and T cell response to different viral infections using NGS-DNA-based platform demonstrated the feasibility of tracking single antigen-specific T-cell clones in tissues to facilitate differential diagnosis [24]. Morris et al. applied Immuno-SEQ approach to characterize and track TCR repertoire in association with tolerance in patients with combined kidney and non-myeloablative bone marrow transplantation (CKBMT) [25]. The authors noted a post transplant reduction in donor-reactive T-cell clones in three CKBMT patients with tolerance; but not in non-tolerant patients or in two with kidney transplant alone on standard immunosuppressive regimens. Additionally, the study showed lower TCRB diversity in non-tolerant subjects compared with that of tolerant subjects [25].

Other techniques such as microarrays [26, 27] were also investigated to identify a molecular signature in kidney biopsy samples with pure TCMR. However, the molecular signature for TCMR had low sensitivity (50%) and low positive predictive value (62%) [12].

Yap et al. [28] showed that a Polychromatic Flow Cytometry and Quantification of sjTRECs could predict long-term allograft dysfunction. The investigators detected a restriction of TCR Vβ diversity associated with the expansion of terminally differentiated effector memory (TEMRA; CD45RA + CCR7 − CD27 − CD28−) CD8+ T-cells. These cells showed an increased expression of perforin, granzyme B, and T-bet, and correlated with the level of CD57 and the ability of CD8+ T-cells to secrete TNF-α and IFN-γ. This TEMRA was detected in patients who later on experienced kidney dysfunction.

Our data confirmed that patients with TCMR had significant increase in blood FOXP3, perforin, granzyme and the T-cell sub-types CD4 and CD8 comparing to those without rejection. Previously, one-step real-time PCR method was utilized as a non-invasive tool to detect the expression of the T-cell activation markers, granzyme B, perforin, and HLA-DRA in PMC [29]. Heng et al. showed that the probability of developing acute rejection in kidney transplant recipients increased from 15% to 73% when both granzyme B and perforin tests were positive, and was reduced to 2% if both were negative [5]. Others also showed that granzyme A mRNA was significantly higher in subclinical and clinical TCMR compared to patients with stable grafts or those with tubular necrosis with 80% sensitivity and up to 100% specificity. Granzyme B and perforin mRNA levels could significantly discriminate acute rejection from stable or tubular necrosis [30, 31].

Unlike previous studies that utilized genomic DNA-based NGS to characterize the TCR repertoire, our study is the first to utilize NGS techniques in detecting TCMR by analyzing mRNA obtained from blood and kidney biopsy samples in a prospective study of kidney transplant recipients. Furthermore, examination of this repertoire in the graft shed a light on the function of the infiltrated T-cells involved in TCMR. Our data confirmed the correlation of upregulation of T-cell markers in the blood with the pathological changes of TCMR. We acknowledge that our findings are limited by the small number of patients who developed TCMR during the study period. Therefore, a follow-up study would be essential to assess delayed TCMR and long term outcome. Nevertheless, our findings constitute a novel tool in utilizing non-invasive next generation T-cell sequencing in the diagnosis of TCMR and in further understanding the T cells characterization as an immune response in the renal transplant in comparison to conventional tools.

## Conclusions

In addition of the potential role of the NGS methods in the diagnosis of TCMR, using these techniques in identifying different specific T-cells clonotypes that correlate with different types of pathogenesis may lead to the discoveries of new immunosuppressions and other T-cell targeted therapies.

## Abbreviations

APCs: Antigen presenting cells
CKBMT: Combined kidney and non-myeloablative bone marrow transplantation
CKD: Chronic kidney disease
CPT: Cell Preparation Tubes
ESRD: End stage renal disease
GVHD: Graft versus host disease
IFTA: Interstitial fibrosis and tubular atrophy
IMGT: ImMunoGeneTics information system
MHC: Major histocompatibility complex
miH: Minor Histocompatibility
NGS: Next generation sequencing
SE: Standard error
TCMR: T-cell-mediated rejection
TCR: T-cell receptor
